# Moderating Role of Self-Esteem Between Perceived Organizational Support and Subjective Well-Being in Chinese Nurses: A Cross-Sectional Study

**DOI:** 10.3389/fpsyg.2019.02315

**Published:** 2019-10-11

**Authors:** Mingli Yu, Shihan Yang, Tian Qiu, Xuege Gao, Hui Wu

**Affiliations:** ^1^Department of Social Medicine, School of Public Health, China Medical University, Shenyang, China; ^2^Department of Mathematics, University of California, San Diego, San Diego, CA, United States

**Keywords:** subjective well-being, perceived organizational support, self-esteem, nurse, moderating effect

## Abstract

**Purpose:**

Nurses are undertaking tremendous physical and psychological pressure, which may reduce their subjective well-being (SWB). This study is aimed to identify the relationship among perceived organizational support (POS), self-esteem, and SWB, and to further explore whether self-esteem could be a moderator in the association between POS and SWB in Chinese nurses.

**Materials and Methods:**

This study was conducted in Liaoning Province in China, in 2018. 606 valid questionnaires were collected. Demographic factors, job conditions, the Index of Well-Being, Survey of Perceived Organizational Support (SPOS), and Rosenberg’s Self Esteem Scale (RSES) were included in each questionnaire. The association among POS, self-esteem, and POS × self-esteem interaction with SWB were examined by hierarchical multiple regression analysis. The interaction was visualized by using simple slope analysis.

**Results:**

Average score of SWB in Chinese nurses was 8.27 ± 2.64. Being married was positively related to SWB, while having longer work time per week, having night shift and dissatisfied with nurse-patient relationship were correlated with lower SWB. POS and self-esteem were important factors for SWB. Self-esteem could moderate the relationship between POS and SWB. When self-esteem was higher, POS had a greater effect on SWB.

**Conclusion:**

Subjective well-being of nurses in the study was at a low level. Self-esteem could moderate the association between POS and SWB. More interventions related to POS and self-esteem will be helpful to improve SWB among nurses.

## Introduction

Subjective well-being (SWB), usually called “happiness,” is defined as people’s perceptions, evaluations, and satisfactions about their lives (Campbell et al., 1976). As a fundamental and basic human concern, SWB is usually measured in two ways: happiness and life satisfaction (Steel et al., 2008; Welsch, 2009; Soukiazis and Ramos, 2016). SWB has been widely studied by scholars in recent decades (Costa and Mccrae, 1980; Dirksen, 1990; van Campen and Iedema, 2007; Pu et al., 2015). A large amount of literature suggests that SWB plays an important role in maintaining mental and physical health, and can reduce the occurrence of depressive symptoms and suicidal tendency, relieve psychological stress, promote healthy lifestyles, alleviate the physical pain of patients, thus reducing the incidence of diseases and prolonging life-span (Baiden et al., 2016; Lucas-Molina et al., 2018; Martin-Maria et al., 2018; Tey et al., 2018; Furrer et al., 2019; Kaiser et al., 2019; Steptoe, 2019). SWB is also inseparable from some positive psychological factors, such as hope and optimism, and can even be a key predictor of quality of life (Bennett et al., 2015; Kaye-Tzadok et al., 2019). Accordingly, SWB still has important research value.

Since the concept of SWB was proposed, there have been many studies on the elderly, students and patients, but few studies have been conducted on nurses or other occupational groups (Lee et al., 2004; Kong et al., 2013b; Kim et al., 2017). In recent years, the mental health status of Chinese nurses has been widely concerned. Due to heavy workload and high work pressure, nurses are prone to negative emotions such as nervousness, irritability, anxiety, or depression symptoms (Zhang et al., 2018; Dai et al., 2019; Lu et al., 2019). In China, nurses are short-staffed and have to undertake more nursing work, which may easily lead to occupational stress and turnover tendency, affecting nurses’ work engagement and quality of nursing services (Wu et al., 2010a; Wang et al., 2017). Unlike developed countries, the nurse-patient relationship may be more tense in China, which may cause mental health problems for nurses and affect SWB (Wu et al., 2010a; Graham et al., 2017). Some studies have shown that the SWB of nurses is lower than that of the general population (Oates et al., 2017; Oates, 2018). Therefore, we believe that the level of SWB of Chinese nurses is worrying and deserves a wide range of attention.

Based on previous literature, several demographic and job characteristics were suggested to be associated with SWB. Piotrkowska et al. (2019) reported that life satisfaction of nurses increased with age and shift-working day and night reduced life satisfaction. Zhou et al. (2015) found the association between higher educational level, being married/cohabitation and higher level of SWB. Another study reported working too many hours per week reduced SWB of Chinese female caregivers (Chen et al., 2019). The influence of income on SWB was also demonstrated (Zhang et al., 2008). Liu et al. (2018) suggested that nurse’s years of experience might be related to burnout, job satisfaction and so on, so we speculated the years of experience may have an impact on SWB. Additionally, because the current nursing care failed to meet the patients’ expectations, the patients often experienced dissatisfaction, which led to frequent nurse-patient conflicts in China. Tense nurse-patient relationship might induce depressive symptoms and occupational stress in nurses (Wu et al., 2010a; Gao et al., 2012b), which was likely to be an influence factor of SWB.

Perceived organizational support (POS) was defined as employees’ subjective perceptions of whether their contributions and health are valued and cared for by the organization (Eisenberger et al., 1986; Chiang and Hsieh, 2012). As an important positive psychological factor, POS is beneficial to nurses’ psychological well-being (Pahlevan Sharif et al., 2018). Liu and Liu (2016) reported that higher POS could increase intention to remain in Chinese nurses. For Chinese nurses, POS might also be a protective factor for burnout and improve the level of engagement (Cao et al., 2016; Wang et al., 2017). Several studies revealed that organizational support played an important role in coping with negative psychology such as stress and depressive symptoms at work (Panaccio and Vandenberghe, 2009; Hao et al., 2016; Wolff et al., 2018). Studies in general and professional groups reported that perceived stress, depression or anxiety symptoms adversely affected SWB (Atanes et al., 2015; Cheng, 2017; Malone and Wachholtz, 2018). POS probably can serve as a protective factor of SWB. But no study directly explored the relationship between POS and SWB among Chinese nurses. What’s more, nurses’ POS was often at a low level in real life (Robaee et al., 2018). If possible, we also tried to explore some positive psychological resources to reduce the dependence of POS for improving SWB.

As a personality trait, self-esteem is used to describe people’ viewpoints about how they respect or accept themselves and their sense of self-worth (Rosenberg, 1965; Lou et al., 2011). Studies have revealed that self-esteem has a protective effect on nurses’ mental health, which can help to fight work-related stress and depression (Shimizu et al., 2004; Lee et al., 2013). The positive effect of self-esteem on SWB or life satisfaction has been discussed by many studies in other groups, such as students, the elderly, breast cancer patients or homosexuals (Kong et al., 2013b; Hu et al., 2016; Tan et al., 2016; Tian, 2016; Cobo-Cuenca et al., 2019). In the study of SWB or life satisfaction, the mediating role of self-esteem was often explored. Researches on Chinese college students presented self-esteem mediated the relationship between social support and life satisfaction (Kong and You, 2013a; Kong et al., 2015). Intergeneration social support might influence life satisfaction through self-esteem among the elderly in China (Tian, 2016). Lu et al. (2015) suggested that self-esteem can serve as an intermediate variable in perceived physical appearance and life satisfaction in Chinese deaf and hearing adolescents. In fact, self-esteem can also serve as a moderator to moderate the strength of the relationship between two variables. Self-esteem could reduce the positive correlation between body-related guilt and the frequency of depressive symptoms among people with higher self-esteem (Brunet et al., 2019). Higher self-esteem might attenuate the effect of stressful life events on non-suicidal self-injury and depression for left-behind children (Lan et al., 2019). Nevertheless, the moderating effect of self-esteem has not been widely and deeply explored. What’ more, there is also a significant correlation between self-esteem and organizational support in Chinese nurses (Liu and Liu, 2016). Based on the above literature, if self-esteem can strengthen the correlation between POS and SWB, it will provide another important clue to improve nurses’ SWB in the current professional environment.

Accordingly, this study aimed to describe the level of SWB in Chinese nurses and identify the association among POS, self-esteem and SWB, and to further explore whether self-esteem could be a moderator in the association between POS and SWB in Chinese nurses.

## Materials and Methods

### Ethics Statement

This study process conformed to ethical standards and was approved by the Institutional Review Board of China Medical University. The written informed consent was collected from each subject who volunteered for this research. In order to protect participants’ privacy, the data obtained from them was kept confidential and anonymous.

### Study Design and Data Collection

This multicenter, cross-sectional study was conducted in Liaoning Province, China in 2018. A multi-stage stratified random sampling method was chosen to recruit participants. Initially, Liaoning Province contains five districts: eastern, southern, western, northern and central regions. Two tertiary hospitals with more than 500 beds were chosen randomly from each region, for a total of 10 hospitals. Because male nurses had less than one percent of Chinese nurses, our research was aimed at female nurses (Tian et al., 2009). Then 70 female nurses were randomly selected from the selected hospitals, for a total of 700 nurses. Nurses were excluded if they worked for less than 1 year. After signing the informed consent form, each participant was given a self-administered questionnaire. Then, 606 valid questionnaires were collected by us, with an effective rate of 86.6% eventually.

### Measurement of Subjective Well-Being

Subjective well-being was measured with the Index of Well-Being (IWB) developed by Campbell et al. (1976). The IWB scale consists of two parts, containing nine items. It can be utilized by individuals to self-measure their current sense of well-being. The first part is the index of general affect (weight of 1) with eight semantic differential items, and each item measures different levels of perception. For example, boring to interesting equals 1–7 points. The second part is a single item that measures overall life satisfaction (weight of 1.1), with seven options resulting a score of 1–7 (1 = complete dissatisfaction, 7 = complete satisfaction). The total score is calculated by the average of index of general affect + life satisfaction × 1.1. The total score is between 2.1 and 14.7. A higher score means higher SWB. The Chinese version of SWB has been widely used with good reliability and validity (Xu et al., 2016; Dong and Ni, 2019). The Cronbach’s α coefficient of the IWB in this study was 0.969.

### Measurement of Perceived Organizational Support

Perceived organizational support was measured by Survey of Perceived Organizational Support (SPOS), compiled by Eisenberger et al. (1986). The Chinese version of SPOS was translated by Ling et al. (2006). The 7-point Likert-type scale includes nine items. SPOS comprises two reverse questions (even if employees try to do the best job they can, the work unit does not notice it; the work unit rarely cares about employees). The total score is from 9 to 63, with higher score indicating higher POS. The Chinese version of SPOS has been proved to have good reliability and validity (Liu et al., 2013; Hao et al., 2016). In our study, the Cronbach’s α coefficient was 0.952.

### Measurement of Self-Esteem

The Chinese version of Rosenberg’s Self Esteem Scale (RSES) (Rosenberg, 1965) has been widely used among Chinese (Shi et al., 2015; Sang et al., 2017). The scale is a ten-item measure. The score for each item is from 1 (strongly disagree) to 4 (strongly agree). Of these, the 3, 5, 8, 9, and 10 items are reverse scoring questions. The higher the total score, the higher the level of self-esteem. In this study, Cronbach’s α coefficient was 0.956.

### Measurement of Demographic Characteristics

Three demographic characteristics were collected for this study, including age, marital status, and educational level. Marital status was divided into two categories: “married/cohabitation” and “single/divorced/widow/separated.” Options for educational level included “junior college and below” and “college and above.”

### Measurement of Job Conditions

Self-designed questions were utilized to assess five job factors, including weekly work time, night shift, monthly income, nurse-patient relationship, and years of experience. Weekly work time was divided into “≤40 h/week” and “>40 h/week.” Night shift was divided into “yes” or “no.” Monthly income (RMB) was categorized as “≤5000 yuan” and “>5000 yuan.” Nurse-patient relationship was evaluated by the question “How often have you been dissatisfied with the nurse-patient relationship at work?” with five possible answers: never, rarely, sometimes, frequently and always. The response was further divided into “moderate dissatisfaction” (never/rarely/sometimes) or “high dissatisfaction” (frequently/always) according to Wu et al. (2010b).

### Statistical Analyses

All the statistical analyses were performed with IBM SPSS Statistics 21.0 (IBM, Asia Analytics Shanghai), with two-tailed probability value of <0.05 considered to be statistically significant. The *t*-test was used to test group differences of continuous variables. Correlation of continuous variables was detected using Pearson correlation analysis. Hierarchical multiple regression was applied to examine the association among POS, self-esteem and SWB as well as to explore the moderating effect of self-esteem in the relationship between POS and SWB. All variables related to SWB in univariate analysis (*P* < 0.05) were adjusted. In step 1, potential control variables were added. POS and self-esteem were entered in step 2. The product of POS and self-esteem was added in step 3. If the interaction effect was statistically significant, simple slope analysis was conducted to visualize the interaction term. In the present study, the Variance Inflation Factor (VIF) values < 10, which indicated that multicollinearity was not an issue in the estimate.

## Results

### Demographic Characteristics and Job Conditions

In this study, the mean score of SWB of the nurses was 8.27 ± 2.64 (mean ± SD). Demographic and job condition variables of participants and group differences on SWB were displayed in Table 1. Of these participants, there were 75.1% of nurses who had married, and 81.0% of them had an educational level of college and above. The percentage of nurses working more than 40 h per week was 53.3%, and 66.5% of nurses had night shifts. Nearly 82.0% of the nurses earned 5,000 yuan or more a month. About twenty percent of nurses were highly dissatisfied with nurse-patient relationship. In addition, the variables including marital status, weekly work time, night shift, and nurse-patient relationship were significantly associated with SWB. Nurses’ score of SWB with an unmarried, divorced, widow, and separated status was lower than that of nurses with a married or cohabited status (*P* < 0.05). With regard to job condition variables, the score of SWB among nurses working more than 40 h per week was significantly lower than that of nurses who worked ≤40 h per week (*P* < 0.05). The score of SWB among nurses who had night shift was significantly lower than those who didn’t have (*P* < 0.05). What’s more, nurses with high dissatisfaction toward nurse-patient relationship had lower levels of SWB than those with moderate dissatisfaction (*P* < 0.001).

**TABLE 1:** Demographic characteristics and job conditions of the study subjects (*N* = 606) and univariate analysis for the factors related to the level of SWB.

**Variable**	**Number of subjects *n* (%)**	**SWB**	***P*-value**
		**(Mean ± SD)**	**(by *t*-test)**
Marital status			0.047
Married/cohabitation	455 (75.1)	8.40 ± 2.67	
Single/divorced/widow/separated	151 (24.9)	7.91 ± 2.52	
Educational level			0.895
Junior college and below	115 (19.0)	8.30 ± 2.72	
College and above	491 (81.0)	8.27 ± 2.62	
Weekly work time			0.012
≤40 h/week	283 (46.7)	8.57 ± 2.80	
>40 h/week	323 (53.3)	8.02 ± 2.46	
Night shift			0.015
No	203 (33.5)	8.65 ± 2.79	
Yes	403 (66.5)	8.08 ± 2.54	
Monthly income (yuan)			0.599
≤5000	109 (18.0)	8.15 ± 2.70	
>5000	497 (82.0)	8.30 ± 2.63	
Dissatisfaction with nurse-patient relationship			<0.001
Moderate dissatisfaction	484 (79.9)	8.52 ± 2.73	
High dissatisfaction	122 (20.1)	7.32 ± 1.99	

### Correlations Among Continuous Variables

Table 2 presented the correlations among age, years of experience, SWB, POS, and self-esteem. The level of SWB was positively correlated with POS and self-esteem. Self-esteem was positively correlated with POS.

**TABLE 2:** Correlations among continuous variables.

**Variable**	**Mean ± SD**	**1**	**2**	**3**	**4**
(1) Age	32.71 ± 5.97	1			
(2) Years of experience	10.25 ± 6.44	0.967^∗∗^	1		
(3) SWB	8.27 ± 2.64	0.028	0.027	1	
(4) POS	42.14 ± 9.57	−0.009	0.002	0.575^∗∗^	1
(5) Self-esteem	24.59 ± 7.76	0.055	0.052	0.496^∗∗^	0.645^∗∗^

*^∗∗^*P* < 0.01.*

### Hierarchical Regression Analyses

**FIGURE 1:**
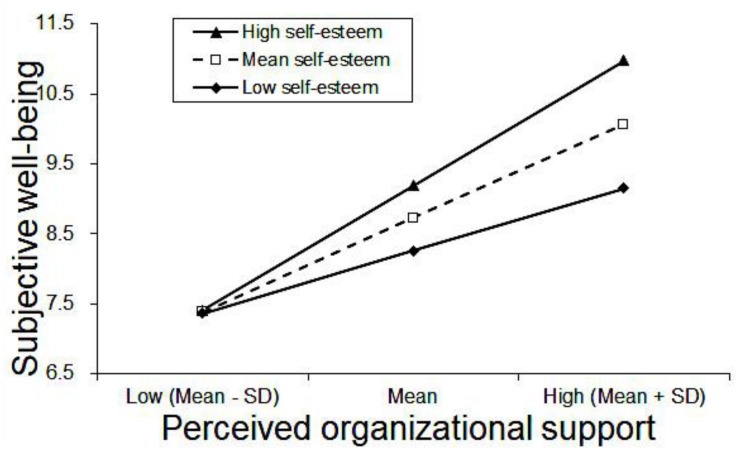
Simple slope plot of the interaction between POS and self-esteem on SWB. The values of POS and self-esteem were centered before regression analysis. Low indicates 1 SD below the mean; high indicates 1 SD above the mean; SD indicates standard deviation.

Table 3 displayed the results of hierarchical regression analyses. First, control variables, including age, marital status, weekly work time, night shift, nurse-patient relationship, significantly explained SWB (adjusted *R*^2^ = 0.047, Δ*R*^2^ = 0.055, *P* < 0.01). The years of experience was highly correlated with age (correlation coefficient = 0.967, *P* < 0.01), and they had multicollinearity (VIF > 16), so years of experience was not added in step 1 (Supplementary Table S1). Weekly work time, night shift and nurse-patient relationship were factors related to SWB. In the second step, POS was found to be significantly and positively related to SWB (β = 0.426, *P* < 0.01), while self-esteem was significantly and positively associated with SWB (β = 0.200, *P* < 0.01). POS and self-esteem improved the model fits of SWB (adjusted *R*^2^ = 0.358, Δ*R*^2^ = 0.311, *P* < 0.01). The POS × self-esteem interaction term was significantly and positively associated with SWB (β = 0.202, *P* < 0.01) in the third step. Simple slope analysis revealed that when self-esteem is higher, the association between POS and SWB becomes stronger. In other words, the impacts of POS on SWB were different in low (1 SD below the mean, β = 0.339, *P* < 0.001), mean (β = 0.508, *P* < 0.001) and high (1 SD above the mean, β = 0.677, *P* < 0.001) levels of self-esteem. The interaction is visualized in Figure 1.

**TABLE 3:** Hierarchical multiple regression results of SWB.

**Variable**	**Step 1**	**Step 2**	**Step 3**
Age	–0.039	–0.008	0.000
Marital status	–0.073	–0.025	–0.015
Weekly work time	−0.085^∗^	–0.026	–0.014
Night shift	−0.094^∗^	–0.048	–0.034
Nurse-patient relationship	–0.173^∗∗^	−0.067^∗^	–0.053
Perceived organizational support		0.426^∗∗^	0.508^∗∗^
Self-esteem		0.200^∗∗^	0.178^∗∗^
Interaction			0.202^∗∗^
*F*	6.920^∗∗^	49.273^∗∗^	50.110^∗∗^
Adjusted *R*^2^	0.047	0.358	0.394
Δ*R*^2^	0.055	0.311	0.036

*Marital status: single/divorced/widow/separated versus married/cohabitation; weekly work time: >40 h/week versus ≤40 h/week; night shift: yes versus no; nurse-patient relationship: high dissatisfaction versus moderate dissatisfaction; ^∗^*P* < 0.05; ^∗∗^*P* < 0.01.*

## Discussion

This study investigated the level of SWB in Chinese nurses. Our results showed that the mean score of SWB among nurses surveyed was 8.27 ± 2.64 (mean ± SD). This score was similar to those reported by some patients (Lee et al., 2004; Unruh et al., 2004; Kim et al., 2017). It was suggested that the SWB of Chinese nurses was at a low level and should be given widespread concern.

Among demographic characteristics and job conditions factors, marital status, weekly work hours, night shift, and nurse-patient relationship were found to have an impact on SWB of Chinese nurses. Nurse, whose marital status is married/cohabitation, may receive more material and spiritual support from their partners to help them cope with life pressure and negative emotions, which leads to higher SWB (Chen and van Ours, 2018). As the previous study presented, with long weekly work time and heavy workload, caregivers are prone to negative emotions, which may impair mental health, decrease the level of SWB (Chen et al., 2019). More than 50% of nurses had weekly work time over 40 h, which exceeded the domestic standard in China. Additionally, long-term night work probably affects nurses’ sleep quality, leading to psychological problems, affecting their perceptions of life, and resulting in reduced SWB (Booker et al., 2019; Dai et al., 2019; Piotrkowska et al., 2019). What’s more, about 20% of nurses surveyed in this study were highly discontent with the nurse-patient relationship. In China, challenges in nurse-patient relationship were on the rise and paid more and more attention (Mok and Chiu, 2005). At present, the shortage of nursing staff and the increased workload of nurses make it difficult for nurses to meet the high demands of patients for nursing services. Coupled with the lack of communication with patients, the relationship between nurses and patients gradually deteriorated. Studies have shown that poor nurse-patient relationship was a risk factor for occupational stress and had negative impacts on the physical and mental health of nurses (Wu et al., 2010a; Gao et al., 2012a, b). This may be the reason why the nurses who are highly dissatisfied with the nurse-patient relationship have lower SWB. However, in this study, the relationship between age, educational level, monthly income, years of experience and SWB was not found in univariate analyses. Understanding the demographic and job status characteristics of SWB can provide a comprehensive model for interventions and policies aimed at nurses to improve their SWB.

The results also revealed that POS was significantly and strongly correlated with SWB. There was a close relationship between POS and job satisfaction among nurses (Gillet et al., 2013). As an important factor of SWB, job satisfaction can obviously and significantly improve the SWB (Gurková et al., 2012; Park et al., 2018). Previous study had similar findings and suggested that employee’s perceived social support has a direct impact on SWB (Pavlova and Silbereisen, 2012). Organizational support is an important aspect of social support. Nurses’ POS provided by hospitals is a powerful social support. In China, owing to enormous physical and mental pressures in work, nurses were prone to psychological problems such as burnout, turnover intention, occupational stress, etc., usually accompanied by a decreased sense of organizational support (Wang et al., 2017; Liu et al., 2018), which may reduce nurses’ SWB.

In this study, there was a positive association between self-esteem and SWB. Additionally, self-esteem was found to moderate the association of POS with SWB. Simple slope analysis revealed that when self-esteem was higher, the effect of POS and SWB was stronger. Based on the vulnerability model, individuals with low self-esteem are prone to social avoidance, which may hinder POS (Orth et al., 2008). With higher self-esteem, people are more confident in life, will face difficulties bravely and have a strong sense of self-worth (Rosenberg, 1965). In addition, people with higher self-esteem have a stronger desire to protect their own self-esteem and be respected by others, organizations and society (Baumeister et al., 2003). Therefore, nurses with higher levels of self-esteem may have stronger desire to be recognized and respected by the organization, then the influence of organizational support on SWB is increased.

In practice, nurses have fewer opportunities to communicate effectively with managers due to busy work, and often accompanied by problems such as occupational stress (Wang et al., 2017), which may result in lower POS and affect their SWB. Here are some suggestions. First of all, nurse managers should improve the allocation of human resources, make reasonable arrangements, pay attention to the mental health and family life of nurses, carry out psychological counseling activities and positive psychology training to improve their SWB. Secondly, nurses can make psychological adjustments themselves, actively seek support from family members, colleagues and leaders and arrange work and life more reasonably. At the same time, training to enhance nurses’ self-esteem, such as assertiveness training (Shimizu et al., 2004), if combined with the above mentioned, will significantly improve the SWB of nurses.

Limitations of this study should be revealed. First, the cross-sectional design didn’t allow us to derive causal relationship between the study variables. Second, participants were from tertiary hospitals with more than 500 beds, where working environment may differ from that of small clinics, thus limit its extrapolation to nurses who work in other types of medical facilities. Third, psychological variables were measured using self-report questionnaires, which may cause recall bias and response bias. We have tried to reduce these biases by using the IWB, SPOS, and RSES, whose appliance in China has been well verified. Finally, we only studied female nurses. We or other scholars can extend this research to male nurses in the future.

## Conclusion

In summary, SWB of nurses in the study was at a low level. Both POS and self-esteem were positively associated with SWB. Self-esteem could strengthen the association between POS and SWB. In addition to giving nurses more care and help in the work, self-esteem intervention should be integrated to help enhance nurses’ SWB.

## Data Availability Statement

The datasets generated for this study are available on request to the corresponding author.

## Ethics Statement

The studies involving human participants were reviewed and approved by the Institutional Review Board of China Medical University. The patients/participants provided their written informed consent to participate in this study.

## Author Contributions

MY contributed to all the processes, including analyzing the data, writing the original draft, making figures and tables, and revising the manuscript. SY, TQ, and XG helped to polish the manuscript and checking the data. HW contributed to the writing of the manuscript.

## Conflict of Interest

The authors declare that the research was conducted in the absence of any commercial or financial relationships that could be construed as a potential conflict of interest.
