# Supplementary material for: Moderating Role of Self-Esteem Between Perceived Organizational Support and Subjective Well-Being in Chinese Nurses: A Cross-Sectional Study
Source: Front Psychol. 2019 Oct 11;10:2315. doi: 10.3389/fpsyg.2019.02315 (PMC6798956; doi:10.3389/fpsyg.2019.02315)
Supplement: Supplementary file 1 [file Table_1.docx]

Supplementary Material

## Supplementary Tables S1

Here are the results of using years of experience instead of age in hierarchical multiple regression. There is no obvious difference between the results of adding years of experience into the regression and adding age into the regression. Because the years of experience was highly correlated with age (correlation coefficient = 0.967, *P*<0.01), and they had multicollinearity (VIF > 16), so in our article, only one of them, age, was added to the hierarchical multiple regression.

| **Variable** | **Step 1** | **Step 2** | **Step 3** |
| --- | --- | --- | --- |
| Years of experience | -0.045 | -0.018 | -0.008 |
| Marital status | -0.073 | -0.027 | -0.017 |
| Weekly work time | -0.085* | -0.026 | -0.014 |
| Night shift | -0.097* | -0.052 | -0.037 |
| Nurse-patient relationship | -0.173** | -0.067* | -0.053 |
| Perceived organizational support |  | 0.426** | 0.508** |
| Self-esteem |  | 0.199** | 0.178** |
| Interaction |  |  | 0.202** |
| *F* | 6.962** | 49.312** | 50.120** |
| Adjusted *R^2^* | 0.047 | 0.359 | 0.394 |
| Δ*R^2^* | 0.055 | 0.311 | 0.036 |

**Hierarchical multiple regression results of SWB (years of experience other than age)**

Note: marital status: single/divorced/widow/separated versus married/cohabitation; weekly work time: >40 h/week versus ≤40 h/week; night shift: yes versus no; nurse-patient relationship: high dissatisfaction versus moderate dissatisfaction; *indicates *P*<0.05; ** indicates *P*<0.01
